# Practical Illustrations of the Remedial Efficacy of a Very Low or Anæsthetic Temperature in Cancer

**Published:** 1851-02

**Authors:** James Arnott

**Affiliations:** Physician to the Brighton Dispensary


*Practical illustrations of the Remedial Efficacy of a very low or Anæsthetic Temperature.—1. In Cancer.* By James Arnott, M. D., Physician to the Brighton Dispensary.—Having already given an account of the antiphlogistic and anodyne properties of a very low or benumbing temperature in the treatment of erysipelas, headache, and other cutaneous and neuralgic affections, as well as of its utility as an anæsthetic, in many surgical operations, in lieu of the inhalation of ether or chloroform, I proceed, on the present occasion, to lay before the profession some details respecting other important applications of the same valuable therapeutical agent. I shall show, in the first place, by a report of a case of cancer treated by an anæsthetic temperature or congelation, that it furnishes us with a perfect means of relieving the pain of that dreadful disease, without producing the stupefaction and disturbance of the system that attends the use of narcotics; and that, instead of precipitating the unfortunate patient's fate, like these, congelation, by arresting the accompanying inflammation, and perhaps destroying the vitality of the "cancer cell," is not only calculated to prolong life for a great period, but may, not improbably, in the early stage of the disease, exert a curative action.

If it be objected that the case to be reported is insufficient evidence of this property of congelation, I would reply, that the unequivocal, immediate, and long-enduring relief from suffering which it shows to have been effected by each of numerous applications, during a period of eight months, together with the restoration of the patient's general health, in consequence of the arrest of the local disease, leave no doubt on this point, and render a single case hardly less satisfactory than a multitude would be. Nay, one such application of the remedy as has been recently made in the Middlesex Hospital, by which long-continued and severe pain was immediately and completely relieved, for a period of fourteen days, (which period had elapsed without any recurrence of pain when I last saw the patient,) would almost be sufficient evidence; corroborated, as such a fact is, by the equally striking effects of the same remedy in other painful diseases.

As the subject will probably, be entirely new to many readers of this paper, it may be proper to give a brief account of the agent whose effects in cancer it is its purpose to describe. The very low or anæsthetic temperature that is used remedially as a local application to inflamed or painful parts, is produced by what are termed frigorific mixtures, or combinations of pounded ice and various salts, which, in dissolving, reduce the temperature below the degree of zero of Fahrenheit's thermometer, or more than thirty degrees lower than any temperature hitherto employed in medicine. The application of such a mixture to the skin, or mucous membrane, causes little sensation of any kind, as the part soon becomes benumbed, and the slight tingling or smarting produced (which is seldom so great as to be complained of by the patient,) is more allied to the sensation of heat than of cold. If the frigorific differs from cold water or ice in the sensations it produces, it differs from them still more in its physiological and remedial effects. There is in fact, no greater resemblance between the effects of different low degrees than there is between different high degrees of temperature;—as, for example, between the soothing heat of fomentations, and the scalding heat that is occasionally resorted to as a powerful stimulant, or the still higher degree which, when communicated by an iron, is often used as an escharotic.

In investigating the pretensions of a new remedy, we require evidence, not only of its power of palliation or cure, but of its general safety. Large and repeated doses of opium or morphia, or copious bleedings unquestionably alleviate the pain from cancer, but it is well known from their effects in this, as well as in other diseases, that they as certainly prove injurious to the patient's constitution, or shorten his life. Now, there is no deficiency of evidence respecting the safety of the remedy which I have to propose as their substitute. Congelation has already been employed, thousands of times, in other inflammatory and painful diseases, without the slightest injury on any one occasion. The prejudice against this remedy on account of its having hitherto been only known in its uncontrolled agency, or as a cause of disease, was not extraordinary, as a similar prejudice has existed against many other powerful agents, which, before they were employed medicinally, had only been known as noxious to the animal economy. Dr. Paris adverts, in severe terms, to this prejudice in his account of the opposition from it to the medical use of prussic acid, saying that it would be just as reasonable to object to the nse of the knife in surgery because when uncontrolled and unguided by the head and hand of the operator, it is capable of doing deadly injury. To condemn medical congelation because the vitality of exposed parts of the body has been lost by long exposure to severe cold, would be as absurd as to condemn medical electricity because persons have been killed by lightning. Nor is another theoretical objection better founded, that reaction must be the consequence of minor degrees of congelation. Instead of reaction following remedial congelation, there is the very opposite condition, insomuch that parts cut in operations after anæsthesia and congelation have been produced by a low temperature, have invariably healed more quickly than under ordinary circumstances. The vessels of the part appear to be rendered incapable, for a long period, of assuming such a degree of morbid excitement as would materially interfere with the healing process.

I cannot perhaps, more forcibly illustrate the safety with which congelation may be used in medicine, than by stating that, very lately, two young ladies, resident in London, were, after having been for some time under my care, themselves employing it—one, every other day, for suppressing the nascent pustules of an acnoid eruption on the face; the other to remove a nævus from the same locality. Were there any danger of the skin receiving the slightest blemish from regulated congelation, I should not thus have left its application to so conspicuous a part in their own hands.

But after the removal of the prejudices and misunderstandings just adverted to, another question is sure to occur to the practitioner: granting the utility and safety of this new antiphlogistic and anodyne remedy, what advantage does it possess over the numerous expedients for the removal of inflammation and pain which we already possess, that will compensate for the trouble of applying it, and of learning to apply it properly?

In the first place, it will cure diseases and relieve pains that cannot be cured or relieved by any other known means; and where the same effect may be produced by other means, it is not produced so rapidly. Both as an antiphlogistic and as an anodyne, congelation is much more powerful as respects many diseases than any agent, or combination of agents, possessing similar qualities hitherto employed in their treatment, and which, on that account, have often proved ineffectual. A very low temperature will arrest every inflammation which is near enough to the surface to be accessible to its influence, and totally and permanently remove irritation from the nerves which it can reach.

In the second place, congelation is a safer remedy than most of those which are usually employed for the same purpose. Bleeding often impairs or prostrates the reparative powers; both antimony and mercury occasionally act as poisons; opium stupifies and excites; and events have shown that, as anæsthetics in surgical operations, ether and chloroform are not altogether without danger. Not once, in upwards of two thousand applications which have now been made of it, has congelation caused the least injury.

Other advantages of congelation might be mentioned, but these of its greater certainty, promptitude and safety, must suffice. Cases are every day happening where life is endangered or lost by inflammation that cannot be subdued by bleeding or the ordinary measures without incurring greater hazard from the debility which they occasion, or other injurious effects; and cases of suffering, to which, from some constitutional peculiarity, the ordinary anodynes are inapplicable. That a great desideratum existed here, was strongly evinced by the recent deplorable case of a much lamented statesman, who died from injuries causing inflammation and intense pain. The medical art has never appeared to greater disadvantage than on that melancholy occasion. The inefficient measures resorted to, only showed the indications which the medical attendants were anxious to fulfil, but which, it would seem, they were unwilling to attempt fulfilling by the means in common use. What appeared to be wanting were, an antiphlogistic remedy that would not debilitate, and an anodyne that would not excite.

"The most dreadful disease to which the human frame is liable, is carcinoma; and, perhaps, of all organs affected by it, uterine cancer is the most painful. This is indeed a horrible disease; the patient's sufferings are aggravated by the knowledge that her complaint is incurable, and she languishes under the double torment of excruciating agony and despair. In the treatment of no disease is the medical man's duty so distressing as that of cancer—to witness the increasing sufferings of his patient day after day—to hear at each succeeding visit the same unvarying statement of pain and misery, while he knows too well how poor and inadequate are the resources of his art—while he is sensible that his utmost power will not avail him beyond blunting, by stupefying remedies, the acuteness of bodily anguish, even if he attains that fortunate and desirable advantage—is a task that calls forth the tenderest sympathy which his nature is capable of experiencing."

This graphic description (from Dr. Ramsbotham's lectures on Midwifery,) of the sufferings from cancer of the womb, will, as being very applicable to the case about to be reported, supply the place of a minute detail of symptoms. Only the more essential points will be noticed.

M. R.—— was admitted a patient at the Brighton Dispensary, on the 25th July, 1849. She is of short stature, thin, of sallow complexion, and about forty-two years of age. She had been lately in St. Thomas's Hospital on account of the same disease for which she now sought assistance; and had complained about eighteen months previously to her entering the hospital.

Her principal symptoms were frequent and severe paroxysms of pain, chiefly in the back and hips; a profuse and most offensive discharge, and occasional hæmorrhage from the vagina; and derangement of the digestive organs. On examination, the neck of the womb was found hard and ulcerated.

For six months the usual palliative treatment was pursued, viz: the exhibition of the preparations of opium and the application of leeches. She complained that the opium made her constantly drowsy and unfit for her occupation as a needlewoman: and the pain was, notwithstanding its use, occasionally so severe as to oblige her to rise from bed and roll on the floor of her room.

In January, I determined upon a trial of congelation, having previously made another careful examination of the uterus. The disease had by this time considerably extended; the neck of the womb was now completely destroyed, and there were several warty excrescences in the upper part of the vagina. Congelation was effected by means of a frigorific mixture of two parts of finely-pounded ice and one part of chloride of sodium, introduced through a wide speculum of gutta percha, having the lower part of its upper opening of a cup-like form; and in order that the temperature might be maintained at the requisite low degree, or below zero of Fahrenheit, the dissolved ice was continuously drawn off by a syphon of peculiar construction. This peculiarity principally consists in a large two-necked bottle being connected with, or constituting part of, the long arm of the syphon; and the purpose of it is, that a stream of water may continue to flow along this part of the syphon, and keep up the suction of its upper extremity, notwithstanding any interruption in the supply. A tube of vulcanized india-rubber forms the remaining part of the syphon, with a small glass tube where it enters the speculum, in order that the rising column of liquid may be seen and regulated by a stop-cock.

The success of this application exceeded my expectation. So soon as I had learned to apply the frigorific properly, I was able to give immediate and entire relief, and this has generally continued complete for about a week. The discharge was soon diminished, and became much less offensive, and the tendency to hemorrhage ceased. From twenty to thirty applications of the frigorific have now been made, and scarcely any other remedy has been used. No advance of the disease appears, on examination, to have taken place, and in other respects there is decided improvement. The patient is not so thin; her appetite is tolerably good; she is stronger, and able to occupy herself in the usual household affairs.

She is directed to call whenever the pain returns. The speculum is generally introduced by herself while in the supine position, and she covers her extremities with a sheet before I enter the apartment. The nates are raised, in order that the speculum may be sufficiently upright to contain enough of the frigorific, which has usually been kept applied for a period varying from a quarter to half an hour. There is a slight sensation of smarting produced for a minute or two, and the pain from the disease has generally ceased within the first five minutes. If the womb be now inspected by removing the frigorific from the speculum, the greater part of its visible surface will be found perfectly white and hard. The application is terminated by allowing about a quart of cold water to run rapidly through the speculum and syphon for the purpose of gradually restoring the natural temperature, and washing away any remaing salt.—*London Lancet.*

